# Delineation variable genotype/phenotype correlations of 6q27 terminal deletion derived from dic(6;18)(q27;p10)

**DOI:** 10.1186/s13039-014-0078-3

**Published:** 2014-11-14

**Authors:** Lili Zhou, Chong Chen, Huanzheng Li, Yunying Chen, Xueqin Xu, Xiaoling Lin, Shaohua Tang

**Affiliations:** 1 Department of Genetics, Dingli Clinical Medical School, Wenzhou Medical University, Key Laboratory of Birth Defects, Wenzhou, Zhejiang China; 2 School of Laboratory Medicine and Life Science, Wenzhou Medical University, Zhejiang, China; 3 Key Laboratory of Medical Genetics, Zhejiang, China

**Keywords:** 6q27 Deletion, Trisomy 18p, Mental retardation, Hypotonia, Rearrangement

## Abstract

**Background:**

Terminal deletion of 6q27 produces a rare syndrome associated with unexplained mental retardation, hypotonia, epilepsy, and multiple malformations. Structural brain malformations are consistently observed, including agenesis of the corpus callosum, hydrocephalus, periventricular nodular heterotopia, polymicrogyria, and cerebellar malformations. Here we report a fetal risk assessment of a 27-year-old woman with mental retardation, hypotonia and dysmorphic features at 17 weeks of pregnancy.

**Results:**

Cytogenetic analyses revealed an addition at chromosome 6qter in the mother. Haploinsufficiency of 6q27 to 6qter (1.3 Mb) and trisomy of the entire short arm of chromosome 18 (15.2 Mb) were found using a single nucleotide polymorphism based array. Results were confirmed by molecular cytogenetics and multiplex ligation-dependent probe amplification. The karyotype of the mother was 46,XX dic(6;18)(6pter → 6q27::18p10 → 18pter).arr [hg19]6q27(169,591,548-170,898,549) × 1,18p11.3p10(12,842-15,375,878) × 3.ish dic(6;18)(q27;p10)(RP11-614P3-,RP11-1035E2+,D18Z1+). Deletion of 6q27 was associated with the structural brain malformations, whereas trisomy of 18p had minor clinical effects. The unbalanced rearrangement of chromosome 6 and chromosome 18 was de novo and was not inherited by the developing fetus.

**Conclusions:**

A rare rearrangement between 6q27 and 18p was identified, which led to a de novo 1.3 Mb deletion of 6q27 and a 15.2 Mb duplication of 18p in an adult with mental retardation, hypotonia, epilepsy, and multiple malformations.

## Background

Terminal deletion of 6q27, ranging from 0.4 Mb to 10.8 Mb, produces a rare syndrome with variable clinical features, including mental retardation, hypotonia, epilepsy, and multiple malformations [1-5]. With this syndrome, structural brain malformations are observed consistently, including agenesis of the corpus callosum (ACC), hydrocephalus, periventricular nodular heterotopia (PNH), polymicrogyria, and cerebellar malformations. Previous studies have determined that the critical deletion region for brain malformation is the terminal 1.7 Mb, which harbors four critical genes *THBS2, PHF10, DLL1*, and *C6orf70* [4]. Trisomy18p, on the other hand, is tolerated in humans with mild clinical signs and symptoms. Trisomy 18p can result from unbalanced segmentation, balanced translocation, direct tandem duplication, or sSMC [6-7].

Here, we report a smallest 6q27 deletion with trisomy18p in a 27-year old patient at 17 weeks gestation. The patient presented with severe mental retardation (MR), hypotonia, and other malformations (Table 1). The goals of this report was to elucidate the genotype/phenotype correlation and test the hypothesis that the deletion results from unbalanced chromosome translocation and inheritance.

**Table 1:** **Clinical features of patients with 6q27 deletions on our case compared with those previously reported in the literature**

	**Sirisha et al.,**	**Sirisha et al.,**	**Dupe et al.,**	**Rigon et al.,**	**Striano et al.,**	**Eash et al.,**	**Rooms et al.**	
	**Patient 6**	**Patient 7**	**Patient 4**	**Patient 1**	**Patient 3**	**Patient 1**		
Patient No.	1	2	3	4	5	6	7	8*
Deletion size (Mb)	2.2	1.7	2.2	2.3	3.1	0.4	1.2	1.3
Age at diagnosis	4 months	30 months	Newborn	17 years	4 years	8 years	18 years	27 years
Sex	F	F	F	F	F	M	M	F
Facial dysmorphism								
Hypertelorism	NA	+	NA	_	+	_	NA	+
Broad nasal bridge	NA	+	NA	+	_	NA	NA	+
Ear anomalies	NA	+	NA	_	+	+	NA	_
Midface hypoplasia	NA	_	NA	+	_	_	NA	_
Long philtrum	NA	_	NA	_	_	_	NA	_
Thin upper lip	NA	_	NA	+	_	_	+	_
Palatal abnormality	NA	_	NA	_	_	_	+	_
Neurodevelopment								
Developmental delay	+	+	NA	+	+	+	+	+
Epilepsy	+	_	NA	_	+	+	+	_
Structural brain anomalies/abnormal	+	+	+	+	+	+	+	+
MRI								
Periventricular nodular heterotopia	NA	NA	NA	+	NA	NA	NA	_
Polymicrogyria	NA	NA	NA	NA	NA	NA	NA	_
Corpus callosum anomalies	+	+	NA	_	_	+	NA	+
Hydrocephalus	+	_	+	_	_	+	NA	_
Vertebral or spinal cord malformation	_	_	_	_	_	+	NA	+
Hypotonia	+	+	_	_	_	_	_	+
Head size	MAC	MIC	NA	NA	NA	NA	MIC	NA
Learning difficulties	+	+	NA	+	+	+	+	_
Inheritance	De novo	Not maternal	De novo	NA	De novo	De novo	Maternal	De novo

*Abbreviations*: *F* female, *M* male, *MAC* macrocephaly, *MIC* microcephaly, *NA* not available.

+ denoted the presence, whereas – denotes the absence of a characteristic, *denotes our case.

## Case presentation

The patient was the product of the first pregnancy of non-consanguineous parents. Both parents and younger brother were healthy, and the family history lacked evidence of mental retardation (MR) and hypotonia. The patient was delivered at 41 weeks gestation. During the entire pregnancy, the mother of the patient felt sick and experienced emesis. Antiabortifacients were taken from 18 weeks of gestation until birth. The patient presented intra-uterine growth retardation; birth weight was approximately 1,500 g (>3SD), and birth length was 40 cm (>2SD); she also exhibited a weak cry and suckling. No apparent congenital abnormalities were noticed, although global developmental delays were observed.

**Figure 1:**
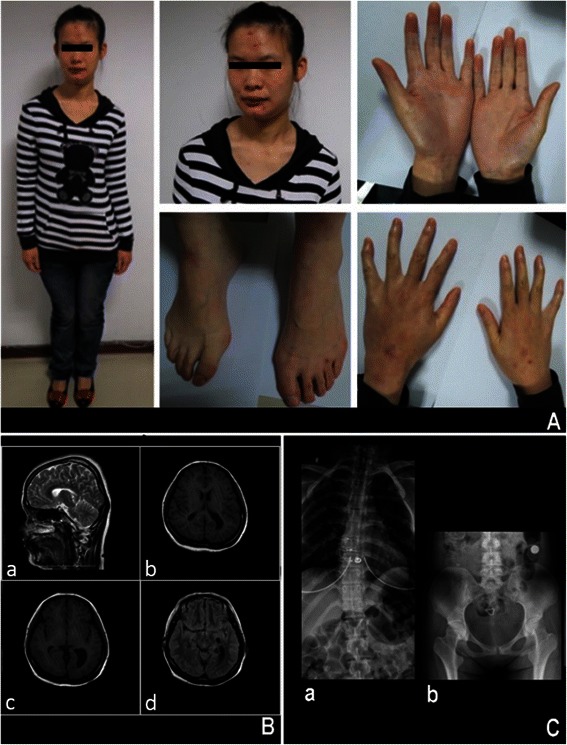
**Clinical and iconography description of the patient. A)** Present patients appearance and facial characteristics. **B)** Brain MRI images show brain structural abnormity: **(a)** T1-weighed sagittal section through the midline, showing corpus callosum hypoplasia; **(b)** T2-weighed axial section showing colpocephaly; **(c)** T2-weighed axial section showing asymmetric lateral ventricles; **(d)** T2-weighed axial section showing choroid fissure cysts on the left. **C)** Radiographic analysis show skeletal structure abnormity: **(a)** Thoracic vertebra showed T8-T10 scoliosis rightward; **(b)** pelvic obliquity.

At age two, the patient had few single words and walked with an unstable gait. At age 27, evaluation revealed an IQ score of 46, hypotonia, prominent forehead, hypertelorism, long philtrum and, slow nystagmus, ataxic gait, joint laxity, bilateral clinodactyly, talipes cavus, scoliosis, pelvic obliquity, genu valgum on right, and femur length discrepancy of 1 cm (Figure 1A).

Brain magnetic resonance imaging (MRI) showed the presence of ACC, colpocephaly, asymmetric lateral ventricles, and choroid fissure cysts on the left (Figure 1B). X-ray showed T8-T10 scoliosis and pelvic obliquity (Figure 1C).

## Results

### HumanCytoSNP-12 BeadChip for CNVs and SNP analyses

**Figure 2:**
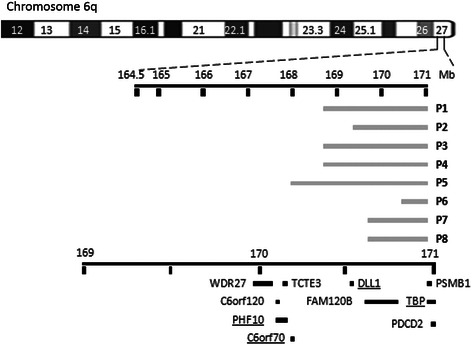
**Size, extent, and genomic content of deletions including 6q27 in cases comparable to the present one.** All patients had structural brain abnormalities. Using this information for mapping of a critical region of brain malformations revealed *C6orf70*, *PHF10*, *DLL1*, and *TBP* as putative candidate genes for structural brain malformations.

**Figure 3:**
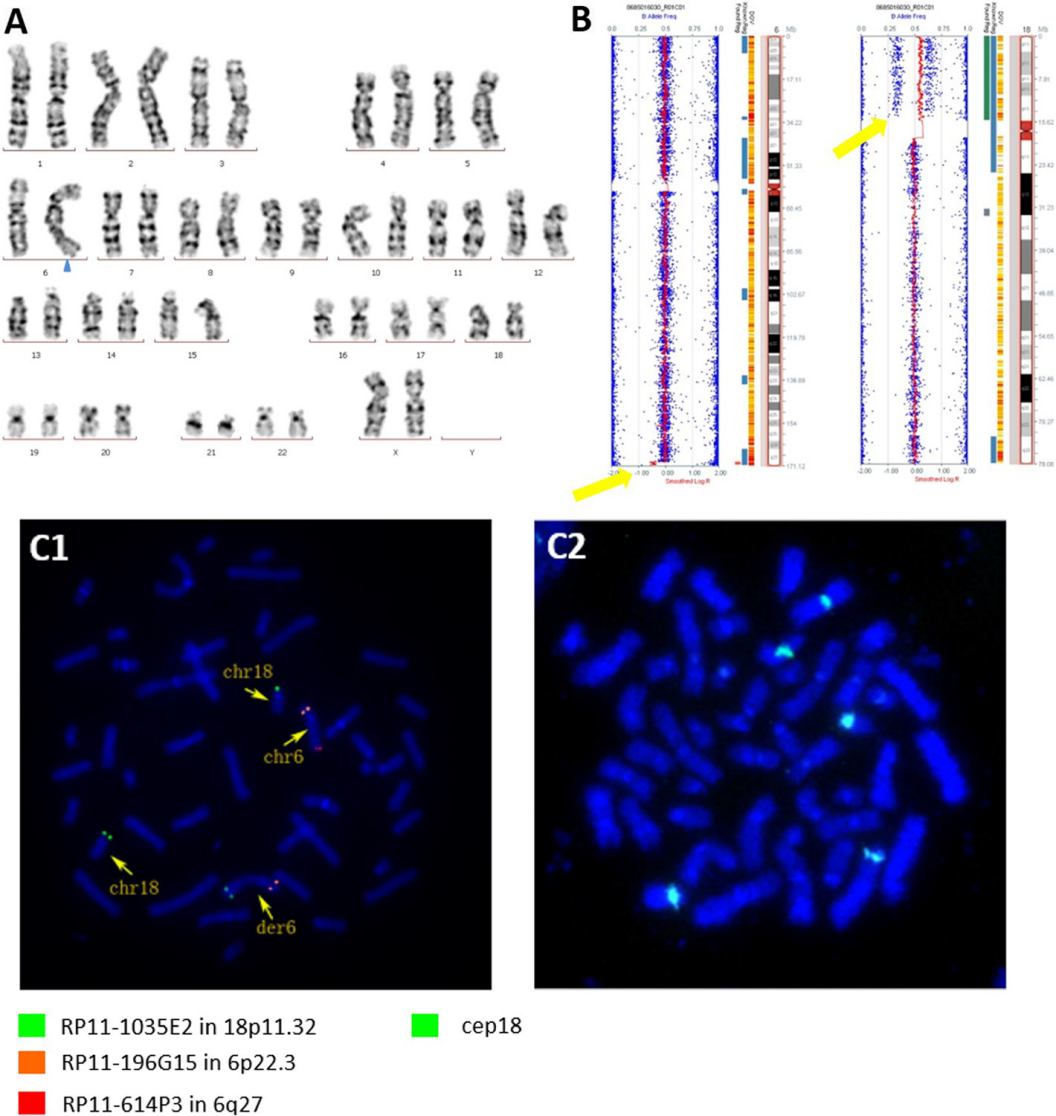
**Cytogenetical and molecular genetical description of the fetus. A)** Cytogenetic analysis revealed a derivative chromosome 6 (blue arrow). **B)** SNP-Array shows deletion of 1.3 Mb at 6q27 to 6qter and a duplication of 15.2 Mb in the short arm of chromosome 18 (yellow arrow). **C)** Fluorescence in situ hybridization (FISH) 1) using BAC-probes RP11-614P3 (=6q27-Red), RP11-196G15 (=6p22.3-Orange), RP11-1035E2 (=18p11.32-Green) demonstrated the deletion of 6q27 and the duplication of 18p11.32; 2) using a centromeric probe D18Z1 (=cep18-Green) gave three signal and indicated that the derivative chromosome 6 is dicentric.

SNP-Array analysis of the present patient revealed a 1.3 Mb de novo deletion from 6q27 to 6qter and a 15.2 Mb de novo duplication including the entire short arm of chromosome 18. The proximal breakpoint in 6q27 mapped to (hg19:169,591,548 to 170,898,549 bp) (Figure 2) and 18p mapped to (hg19:12,842 to 15,375,878 bp) (Figure 3B).

### *Cytogenetic and fluorescence* in situ *hybridization (FISH) analyses*

Chromosome karyotype analyses showed 46,XX,add(6)(q27) (Figure 3A). Her parental and fetal karyotypes were normal. FISH confirmed the deletion of 6q27 and duplication of 18p including parts of the centromere (Figure 3C).

#### Multiplex ligation-dependent probe amplification (MLPA) and real-time qPCR analyses

**Figure 4:**
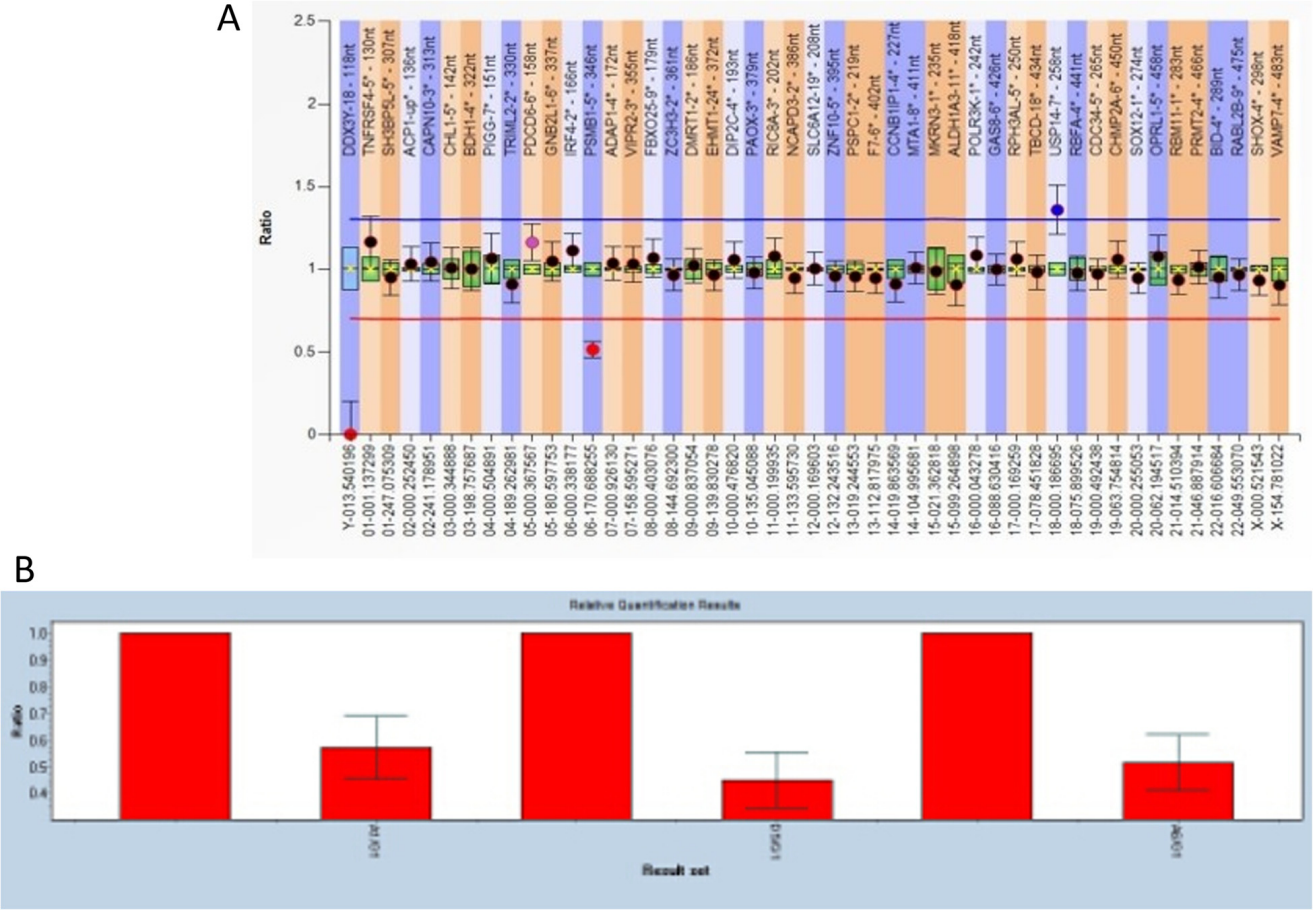
**MLPA and QF-PCR results. A)** MLPA results with probe P036-E2. Probes targeting 18p11.32 (blue) were increased and the signals for 6q27 (red) were decreased, indicating unbalanced translocation with deletion of 6q27 and duplication of 18p; **B)** QF-PCR results with SYBR for gene C6orf70. PCR primer targeted exon 2,exon 12 and exon 18 of C6orf70 and showed the relative quantification ratios were 0.5741, 0.4519 and 0.5163, respectively, indicating the gene is heterozygous deletion.

MLPA P036-E2 was used to confirm the deletion of 6q27 and duplication of 18p (Figure 4A). Meanwhile, haploinsufficiency of the *C6orf70* gene, related to the clinical phenotype of 6q27 deletion syndrome, was confirmed by qPCR (Figure 4B). From these data, the karyotype of the patient was revised to 46,XX,dic(6;18)(6pter → 6q27::18p10 → 18pter).arr [hg19]6q27(169,591,548-170,898,549) × 1,18p13p10(12,842-15,375,878) × 3.ish dic(6;18)(q27;p10)(RP11-614P3-,RP11-1036E2+,D18Z1+).The risk of recurrence for the further pregnancy was 50%.

## Discussion

Here we report a patient with MR in the 17th week of pregnancy. Standard cytogenetic analyses revealed additional chromosomal material at the end of chromosome 6. SNP-Array analyses detected a 1.3 Mb deletion of 6q27-qter and a 15.2 Mb duplication of the entire short arm of chromosome 18. Result from the SNP-Array were confirmed by FISH and MLPA.

Deletion of 6q27, ranging from 0.4 Mb to 10.8 Mb, can result from unbalanced translocation or isolated deletion and produce a rare syndrome [1-5]. Partial terminal monosomy of 6q27 produces brain structural abnormalities, dysmorphic features, developmental delay, epilepsy, learning difficulties, and hypotonia. Patients with distal 6q27 deletions have been described, revealing variable brain phenotypes including agenesis of the corpus callosum (ACC), periventricular nodular heterotopias (PNH), cerebellar malformations, polymicrogyria, and hydrocephalus. Previous studies have shown that terminal 1.7 Mb of 6q27 harbors four genes, *THBS2, PHF10, DLL1*, and *C6orf70* that play critical roles in morphogenesis of the nervous system during embryogenesis [4].

ACC is associated with many syndromes and shows significant variability ranging from complete absence or partial absence to hypoplasia of the corpus callosum. Twelve genomic loci are consistently associated with ACC, including the 6q terminal deletion [8]. Incidence of corpus callosum anomalies for 6q25-27 deletion is approximately 50% to 75%. The subject of this study presented with mild hypogenesis, a short, comma-shaped corpus callosum (Figure 1B). PNH lining the lateral ventricles due to defective neuronal migration and can by asymptomatic or present as small, unilateral or bilateral nodules, or as extensive agglomerates, with or without other brain abnormalities.

*C6orf70* in the 6q27 region is a critical gene expressed during brain development in human and rodents. The gene product is a putative vesicle-associated protein that plays a major role in controlling neuronal migration. Haploinsufficiency or mutation of *C6orf70* was reported to cause PNH [1], and incidence of PNH was 9 out of 12 patients of 6q25-27 deletion. Although PCR analyses confirmed the patient in this report had haploinsufficiency of *C6orf70*, no evidence of PNH was found, suggesting that haploinsufficiency of *C6orf70* for PNH has variable phenotypic expression.

Other brain malformations, such as cerebellar malformation, polymicrogyria, and hydrocephalus, have also been reported in 6q27 deletion cases with variable penetrance and expression. Cerebellar malformations were reported in 10.8% and polymicrogyria in 6.7% of individuals. Interestingly, neither of these conditions were present in the patient characterized in this study, although colpocephaly, asymmetric lateral ventricles, and choroid fissure cysts on the left were present. These results may suggest that the same genes cause various different brain malformations through the same pathway. It is also possible that low resolution MRI may have missed subtle brain malformations.

Mental retardation and seizures are associated with brain malformation in 6q27 deletion patients. The TATA-binding protein *(TBP)* gene in 6q27 seemed the most likely causal mutation [3]. TATA-binding protein, a general transcription factor associates with aggregates in several polyglutamine disorders [9]. Although trisomy18p alone is tolerated in humans and presents as very mild mental retardation [6-7], it is reasonable to speculate that severe MR (IQ = 46) in the subject may result from the combination of *TBP* deletion and trisomy18p.

Hypotonia is a clinical phenotype that affects approximately half of 6q27 deletion patients. Brain malformation may cause this phenotype, and long-lasting repercussions of hypotonia without corrective procedures, include scoliosis, pelvic obliquity, genu valgum, and leg discrepancy. In this case study, chromosome 6 is dicentric, one centromere is from chromosome 6 and the other is from chromosome 18. When human chromosome are dicentrics or multicentrics, faulty alignments may result [10]. Three different ‘predominant’ activation patterns occur. When the distance between two centomeres is close, they are fused and operate together. When the distance between centromeres is 1.4 Mb to 13 Mb, both centromeres are active, and when the distance is over 15 Mb, only one is active [11]. In this case, the distance between the centromeres was estimated to be more than 15 Mb, indicating only one centromere was active.

Assessment of risk for the offspring was a major goal of this study. Results indicated no genetic abnormalities were present in the amniotic fluid, and the fetus was developmentally normal. The baby was born with no complications.

## Conclusions

In conclusion, this study identified a rare rearrangement between 6q27 and 18p, which led to *de novo* 1.3 Mb deletion of 6q27 and 15.2 Mb duplication of 18p in an adult with mental retardation, hypotonia, epilepsy, and multiple malformations. Analyses revealed that the 6q27 deletion contributed to the majority of the patient’s clinical features.

## Methods

### HumanCytoSNP-12 BeadChip for CNVs and SNP analyses

DNA was analyzed with the Illumina Human CytoSNP-12 array to 100 kb resolution following the instructions provided in the Illumina Infinium® HD Assay Ultra manual. The iScan scanner was used to translate electronic signals into digital signals. Initial analyses and quality control were performed using Illumina GenomeStudio software. Copy number variation was determined using the Illumina KaryoStudio software.

### Cytogenetic and FISH analyses

Chromosome preparations were obtained from cultured peripheral lymphocytes and amniotic fluid cells using standard cytogenetic protocols at 320-450 bands resolution. For FISH, probes RP11-614P3 (=6q27-Red), RP11-196G15 (=6p22.3-Orange), D18Z1 (=cep18-Green), RP11-1035E2 (=18p11.32-Green), (JiaHui, China) were used according to standard cytogenetic protocols.

### MLPA and real-time qPCR analyses

P036-E2 probe mix contains a probe designed to detect deletions and duplications for every subtelomeric region. MLPA reactions were performed following the manufacturer’s instructions (Biometra Thermal Cycler, Westburg, Netherlands). Fragment separation was performed using the ABI-3130 sequencer (Applied Biosystems, Foster City, CA), and data was directly analyzed using Coffalyser software. Primers for the *C6orf70* gene were designed for use in real-time qPCR analyses on an ABI 7900 HT fluorescence quantitative PCR instrument (Applied Biosystems, Foster City, CA). Primers sequences and PCR conditions are available by request.

## Consent

Written informed consent was obtained from the patient for publication of this case report and accompanying images.

## Abbreviations

ACC: Agenesis of corpus callosum
FISH: Fluorescence in situ hybridization
MLPA: Multiplex ligation-dependent probe amplification
MR: Mental retardation
SNP-Array: Single nucleotid polymorphism based array
PNH: Periventricular nodular heterotopias
sSMC: Small supernumerary marker chromosomes
