# Erratum to: Delineation variable genotype/phenotype correlations of 6q27 terminal deletion derived from dic(6;18)(q27;p10)

**DOI:** 10.1186/s13039-016-0287-z

**Published:** 2016-10-04

**Authors:** Lili Zhou, Chong Chen, Huanzheng Li, Yunying Chen, Xueqin Xu, Xiaoling Lin, Shaohua Tang

**Affiliations:** 1 Department of Genetics, Dingli Clinical Medical School, Wenzhou Medical University, Key Laboratory of Birth Defects, Wenzhou, Zhejiang China; 2 School of Laboratory Medicine and Life Science, Wenzhou Medical University, Zhejiang, China; 3 Key Laboratory of Medical Genetics, Zhejiang, China

## Erratum

Unfortunately, the original version of this article [1] contained an error. The Acknowledgement section contained the incorrect funding number for Major Program of Ministry of Health, instead of NO.WKJ2011-2-017 it should be NO.WKJ2011-2-019. The corrected Acknowledgment section can be found below.
